# Cancer of the Upper Alimentary Tract and Larynx in Poland and in Polish-born Americans

**DOI:** 10.1038/bjc.1974.86

**Published:** 1974-05

**Authors:** J. Staszewski

## Abstract

Mortality for cancers of the buccal cavity and pharynx, oesophagus and larynx in Poland in 1959-72 was analysed and compared with cancer incidence registered in the selected regions of Poland, with cancer mortality and incidence in other countries, and with mortality among Polish-born migrants to the U.S.A.

The patterns of occurrence of these cancers in Poland appear to be similar to those of other European and American countries, except perhaps for the rather high and still increasing incidence of laryngeal cancer. Among male Polish migrants, however, mortality for these cancers was distinctly higher than either in Poland or among native Americans. This contrast, largest for oesophageal and laryngeal cancer, decreased between 1950 and 1959-61, but only for those aged below 65. Similarity of these shifts with those observed for lung cancer is stressed and explanations are looked for. Factors associated with the studied cancers and outlines for the further studies are discussed briefly.


*Br. J. Cancer* (1974) **29**, 389

### CANCER OF THE UPPER ALIMENTARY TRACT AND LARYNX IN POLAND AND IN POLISH-BORN AMERICANS

J. STASZEWSKI

*From the Institute of Oncology, Gliwice, Poland*

Received 20 September 1973. Accepted 28 January 1974

**Summary.—Mortality for cancers of the buccal cavity and pharynx, oesophagus and larynx in Poland in 1959–72 was analysed and compared with cancer incidence registered in the selected regions of Poland, with cancer mortality and incidence in other countries, and with mortality among Polish-born migrants to the U.S.A.**

**The patterns of occurrence of these cancers in Poland appear to be similar to those of other European and American countries, except perhaps for the rather high and still increasing incidence of laryngeal cancer. Among male Polish migrants, however, mortality for these cancers was distinctly higher than either in Poland or among native Americans. This contrast, largest for oesophageal and laryngeal cancer, decreased between 1950 and 1959–61, but only for those aged below 65. Similarity of these shifts with those observed for lung cancer is stressed and explanations are looked for. Factors associated with the studied cancers and outlines for the further studies are discussed briefly.**

CANCERS of the upper alimentary tract and the larynx have been discussed together here because these sites are likely to be exposed to some of the same carcinogens and, moreover, cancers of these sites are mainly squamous cell carcinomata.

### MATERIAL AND METHODS

The age-specific mortality rates for cancers of the upper alimentary tract and the larynx were computed from unpublished data for the years 1959 through 1972 for Poland. The age-specific cancer mortality rates of Polish-born Americans were available only for 1950 and 1959–61 (see Acknowledgements). The age-adjusted rates were computed using as the standard the " world population " suggested by Segi and Kurihara (1972) and modified by Doll and his colleagues (Doll, Muir and Waterhouse, 1970).

Cancer morbidity data from 4 selected regions of Poland have been compared with similar data from other countries (Doll *et al.*, 1970).

A more detailed description of Polish mortality, morbidity and demographic data (partly described earlier—Staszewski, 1964), and of the background of this study, is being published elsewhere (Staszewski 1974), as also are more data from the Polish cancer registries (Koszarowski *et al.*, 1972).

### RESULTS

### *A. General considerations*

Cancer of the buccal cavity and pharynx accounted for 2·2% of male and 1·0% of female cancer deaths in 1959 in Poland. In 1972 these percentages were, respectively, 3·0 and 1·0. In cancer incidence data from the 4 selected regions of Poland the corresponding percentages ranged from 3·5 for males and 1·1 for females in Warszawa City to 7·2 and 2·1, respectively, in the Kraków region.

For the individual subsites, as defined by the International Classification of Diseases (ICD), the combined mortality data for the period 1961–72 have been used for computation of the rates (figures by age were not available before 1961 for these subsites). Among these subsites, cancer of the lip stands out as the most frequent one in the incidence data, but it does not give the most frequent cause of death (Table I). Such discrepancies between incidence data and mortality are due, at least partly, to the substantial differences in survival existing between these subsites: cancer of the lip is more readily curable than cancer of the tongue, mouth or pharynx.

For all these cancers the incidence and mortality rates were higher in males than in females, particularly in the case of cancer of the lip.

Cancer of the oesophagus was responsible for 3·8% of male and 1·4% of female cancer deaths in Poland in 1959, whereas in 1972 these percentages were 2·8 and 1·1 respectively. In the above mentioned 4 cancer registration areas, the corresponding percentages ranged from 1·7 to 2·8 in males, and from 0·2 to 0·9 in females (see Table II).

Cancer of the larynx was the cause of 1·4% of male and 0·2% of female cancer deaths in Poland in 1959, and 2·1 and

TABLE I.—*Cancer of the Buccal Cavity and Pharynx by Subsites* (*Nos.* 140–148). *Age-Adjusted*/ Average Annual Mortality and Incidence Rates, per* 100,000 *Population by Sex*

| Cancer site and ICD No. 1955 rev. | Mortality 1961–72. Poland, whole country | | Incidence, 1965–66: Warszawa | | Katowice | | Kraków | | For rural areas | |
|---|---|---|---|---|---|---|---|---|---|---|
| | No. | Rate | No. | Rate | No. | Rate | No. | Rate | No. | Rate |
| *A. Males* | | | | | | | | | | |
| Lip (140) | 1500 | 1·0 | 33 | 2·7 | 98 | 2·9 | 166 | 7·2 | 19 | 3·7 |
| Tongue (141) | 578 | 0·3 | 13 | 1·2 | 27 | 1·0 | 18 | 0·7 | 7 | 1·4 |
| Salivary gland (142) | 261 | 0·2 | 6 | 0·5 | 5 | 0·2 | 33 | 1·4 | 5 | 0·9 |
| Mouth—other (143, 144) | 964 | 0·6 | 15 | 1·3 | 5 | 0·2 | 13 | 0·6 | 5 | 0·9 |
| Nasopharynx (146) | 307 | 0·2 | 3 | 0·3 | 9 | 0·2 | 13 | 0·5 | 0 | 0·0 |
| Other pharynx (145, 147, 148) | 2215 | 1·4 | 17 | 1·6 | 23 | 0·8 | 18 | 0·8 | 7 | 1·4 |
| *B. Females* | | | | | | | | | | |
| Lip (140) | 369 | 0·2 | 4 | 0·2 | 12 | 0·3 | 14 | 0·4 | 6 | 0·9 |
| Tongue (141) | 171 | 0·1 | 7 | 0·4 | 20 | 0·5 | 6 | 0·2 | 0 | 0·0 |
| Salivary gland (142) | 189 | 0·1 | 14 | 0·9 | 7 | 0·2 | 28 | 1·0 | 1 | 0·3 |
| Mouth—other (143, 144) | 508 | 0·2 | 6 | 0·4 | 1 | 0·0 | 9 | 0·3 | 2 | 0·3 |
| Nasopharynx (146) | 201 | 0·1 | 3 | 0·2 | 10 | 0·3 | 0 | 0·0 | 1 | 0·1 |
| Other pharynx (145, 147, 148) | 712 | 0·3 | 4 | 0·2 | 12 | 0·3 | 20 | 0·7 | 1 | 0·1 |

* Standard—*World Population* (Doll *et al.*, 1970).

TABLE II.—*Cancer of the Buccal Cavity and Pharynx, Oesophagus and Larynx. Average Annual Age-adjusted Incidence Rates per* 100,000 *Population by Sex,* 1965–66

| | Buccal activity and pharynx (140–148) | | Oesophagus (150) | | Larynx (161) | |
|---|---|---|---|---|---|---|
| | Number | Rate | Number | Rate | Number | Rate |
| *A. Males* | | | | | | |
| Warszawa | 87 | 7·6 | 61 | 5·4 | 88 | 7·8 |
| Katowice | 167 | 5·3 | 115 | 3·8 | 173 | 5·5 |
| Kraków | 261 | 11·2 | 63 | 2·7 | 151 | 6·7 |
| 4 rural areas | 43 | 8·3 | 20 | 4·2 | 31 | 6·4 |
| *B. Females* | | | | | | |
| Warszawa | 38 | 2·3 | 38 | 1·9 | 15 | 0·8 |
| Katowice | 62 | 1·6 | 24 | 0·5 | 15 | 0·4 |
| Kraków | 77 | 2·6 | 16 | 0·5 | 11 | 0·4 |
| 4 rural areas | 11 | 1·7 | 2 | 0·3 | 2 | 0·3 |

* Standard—*World Population* (Doll *et al.*, 1970).

0·2% respectively in 1972. In the 4 cancer registries these percentages ranged in 1965–66 from 3·6 to 4·3 for males and from 0·2 to 0·4 for females. As with buccal cavity and pharyngeal cancers, incidence rates were highest in Warszawa City.

*B. Age*

For each of the cancers under study, incidence and mortality rates increase with age, as do most other cancers, but for the male rates there is seen to be an unusual change in the laryngeal cancer mortality. The mortality among Polish males ceases to increase at about the age of 65 years, and at older ages even decreases (Fig. 1). Among the U.S. native whites a slowing down of the mortality increase with age is also noticeable at about the same age (Fig. 4). The curve of mortality by age for laryngeal cancer among Polish males is similar in shape to that for lung cancer (Staszewski, in the press). The deflection of the curve of mortality by age in males was less pronounced for oesophageal cancer than for laryngeal cancer (Fig. 2), and was not evident for cancer of the buccal cavity and pharynx considered as one site (Fig. 3a), nor for the subsites lip, salivary gland and nasopharynx, but could be noticed in the curve for the remaining subsites (Fig. 3b).

FIG. 1.—Age-specific laryngeal cancer mortality rates per 100,000 population by sex in Poland in 1959–61 and 1970–72.

FIG. 2.—Age-specific oesophageal cancer mortality rates per 100,000 population by sex in Poland in 1959–61 and 1970–72.

FIG. 3.—Age-specific buccal cavity and pharynx cancer mortality rates per 100,000 population by sex in Poland. (a) All subsites, in 1959–61 and 1970–72; (b) subsites lip, salivary gland and nasopharynx, compared with the remaining ones, in 1961–72.

For females the rates are based on smaller numbers and are less stable, especially for laryngeal cancer (Fig. 1–3).

*C. Urban–rural differences*

For all the cancer sites discussed, mortality among Polish men was higher in urban than in rural areas. This differential was highest for laryngeal cancer and lowest for cancer of the buccal cavity and pharynx. It decreased in the 1960s for both sexes, due mainly to an increase in the rural rates (Table III), which may be attributable to the improvement in the diagnosis and certification of causes of death, indicated by a marked decrease in the proportion of deaths certified as due to senility and other ill defined conditions (Staszewski, in the press).

Among females the urban–rural differential was less marked, and for cancer of the buccal cavity and pharynx the rural rates became recently higher than the urban ones.

Comparison of incidence rates reported in Warszawa City and in the 4 rural areas (Table II) corroborates the higher risk of laryngeal cancer in urban—and of cancer of the buccal cavity in rural populations—the latter contrast depending mainly on the higher incidence of the lip cancer in rural areas.

*D. Secular trends*

The male mortality for cancer of the buccal cavity and pharynx increased in Poland up to 1964, then remained virtually unchanged until 1969, and increased again in 1970–72 (Fig. 4). This increase affected all the age groups (Fig. 1). The earlier increase was somewhat faster in the older age groups (aged 65 and over), whereas the later increase concerned

TABLE III.—*Cancer of the Buccal Cavity and Pharynx, Oesophagus and Larynx in Poland,* 1959–61 *and* 1967–69. *Age-adjusted Average Annual Mortality Rates, per* 100,000 *Population, by Urban–rural Residence and Sex*

| Cancer site and ICD No. 1955 rev. | Sex | 1959–61 Urban | 1959–61 Rural | 1967–69 Urban | 1967–69 Rural |
|---|---|---|---|---|---|
| Buccal cavity | M | 3·0 | 1·7 | 3·7 | 3·4 |
| and pharynx (140–148) | F | 0·9 | 0·6 | 0·8 | 1·0 |
| Oesophagus (150) | M | 6·0 | 2·9 | 5·4 | 4·2 |
| | F | 1·7 | 1·0 | 1·2 | 1·1 |
| Larynx (161) | M | 2·8 | 0·8 | 4·6 | 2·8 |
| | F | 0·3 | 0·2 | 0·4 | 0·3 |

* Standard—*World Population* (Doll *et al.*, 1970).
† According to administrative criteria.

more the younger age groups. The pattern of the female rates was similar but less marked; these rates were based on smaller numbers than the male ones.

The male oesophagus cancer mortality increased in Poland before 1964 (Fig. 4). This increase was larger in the older age groups (after 65), which indicates that it was rather a spurious increase due to improved diagnosis in the old. After 1965 a slight decrease in the rates was apparent. Female mortality rates show a similar pattern.

Laryngeal cancer age-adjusted mortality rates increased in males throughout the 1959–72 period (Fig. 4). This increase was more marked in the young age groups. In females the rates are based on small numbers, and are therefore less stable, and the trend is less marked, but a decrease in mortality in the older age groups is apparent after 1965.

FIG. 4.—Time trends in age-adjusted mortality rates per 100,000 population by sex for cancers of the buccal cavity and pharynx, oesophagus and larynx, in the 1959–72 period in Poland.

### *E. Comparison with other countries*

Had Polish mortality figures been included in Segi's compilation of mortality rates in 24 countries for 1966–67 (Segi and Kurihara, 1972), Poland would rank 13th for males and 17th for females for cancer of the buccal cavity and pharynx, 10th for males and 15th for females for oesophageal cancer, and 6th and 7th respectively for laryngeal cancer. The age-adjusted Polish mortality sex ratios for these 3 cancers were 4·0 : 1, 4·1 : 1 and 8·6 : 1 respectively, and were in the middle of the range of the sex ratios reported for these 24 countries. These sex ratios increased in Poland throughout the 1959–72 period, most distinctly for laryngeal cancer and least for oesophageal cancer.

As in most of the 24 countries, mortality for oesophageal cancer changed little in Poland. On the other hand, the increase in mortality from cancers of the larynx and of the buccal cavity and pharynx, observed in Polish males, is relatively large when compared with the trends reported for the 24 countries, in most of which, in the 1950–67 period, mortality did not appreciably change (and in some decreased) and increased to a similar extent in Poland as in France (and for laryngeal cancer also in Italy and in U.S. non-whites).

Analysis of international morbidity data also indicates that Poland occupies an intermediate position for the cancers discussed, except for cancer of the larynx for which Poland has a relatively high rate.

### *F. Polish migrants*

As can be seen from Fig. 5 and Table IV, in 1959–61 mortality rates for cancer of the buccal cavity and pharynx among the Polish-born American males were similar to those among native white Americans, and distinctly higher than in Poland at the same time (but similar to those in Poland 10 years later).

Between 1950 and 1959–61 mortality for this cancer among Polish-born American males decreased largely (by about one-third, Table IV). This decrease, pertaining only to the age groups below 65 years of age (Fig. 6), was larger than the decrease experienced by the native Americans: in 1950 mortality for this cancer among Polish migrants was 19% higher (Haenszel, 1961), and in 1959–61 8% lower than mortality among the native whites at the same periods.

Polish-born American females experienced a little lower mortality than native American females (Table IV).

Oesophageal cancer mortality in males was highest in Polish-born Americans in 1959–61, lower in Poland and lowest in native white Americans (Fig. 5, Table IV).

FIG. 5.—Age-specific mortality rates per 100,000 males for cancers of the buccal cavity and pharynx (Nos. 140–148, ICD, 1955 rev.), oesophagus (No. 150) and larynx (No. 161) in 1959–61, in Polish-born and native white Americans and in Poland.

FIG. 6.—Age-specific mortality rates per 100,000 males for cancers of the buccal cavity and pharynx (Nos. 140–148, ICD, 1955 rev.), oesophagus (No. 150) and larynx (No. 161) in Polish-born Americans, 1950 and 1959–61.

TABLE IV.—*Cancer of the Buccal Cavity and Pharynx, Oesophagus and Larynx. Average Annual Age-adjusted Mortality Rates, per* 100,000 *Population by Sex*

| | | Buccal cavity and pharynx (140–148) | | Oesophagus (150) | | Larynx (161) | |
|---|---|---|---|---|---|---|---|
| | | Number | Rate | Number | Rate | Number | Rate |
| *A. Males* | | | | | | | |
| Polish-born Americans | 1950 | 72 | 6·1 | 131 | 10·7 | 48 | 3·6 |
| | 1959–61 | 225 | 4·2 | 332 | 6·1 | 161 | 2·9 |
| U.S. native whites | 1959–61 | 10249 | 4·6 | 6948 | 3·1 | 4454 | 2·0 |
| Poland | 1959–61 | 798 | 2·3 | 1448 | 4·2 | 603 | 1·7 |
| Poland | 1967–69 | 1561 | 3·6 | 2068 | 4·7 | 1601 | 3·5 |
| Poland | 1970–72 | 2175 | 4·6 | 2150 | 4·6 | 1601 | 3·5 |
| *B. Females* | | | | | | | |
| Polish-born Americans | 1950 | 10 | 1·0 | 12 | 1·1 | 2 | 0·1 |
| | 1959–61 | 30 | 1·0 | 72 | 1·4 | 7 | 0·2 |
| U.S. native whites | 1959–61 | 3360 | 1·3 | 2042 | 0·7 | 516 | 0·2 |
| Poland | 1959–61 | 364 | 0·7 | 620 | 1·3 | 117 | 0·2 |
| Poland | 1967–69 | 568 | 0·9 | 710 | 1·1 | 221 | 0·4 |
| Poland | 1970–72 | 650 | 1·0 | 722 | 1·1 | 171 | 0·3 |

* Standard—*World Population* (Doll *et al.*, 1970).

The difference pertained mainly to age groups over 60.

In 1950 the contrast between Polish-born and native white Americans was even larger than in 1959–61. Between 1950 and 1959–61, mortality from this cancer decreased appreciably in Polish migrants—in the younger (below 60) group by more than half (Fig. 6). In 1959–61 it was only 2·0 times as high as among native whites, whereas in 1950 it was 3·4 times as high (Haenszel, 1961).

At both times the difference between mortality rates of the Polish-born and the native white Americans was larger at the older ages.

Female oesophagus cancer mortality was similar in Poland and among Polish-born Americans, and higher than among American native whites (Table IV).

Laryngeal cancer mortality among males was, in 1959–61, higher among Polish migrants than among the native Americans. This difference was limited to the age groups over 60, whereas below 60 the rates in both these groups were similar. Mortality in Poland was at that time slightly lower than in the United States native whites (Table IV, Fig. 5).

The mortality rate for the age group 55–64 of the Polish-born Americans was lower in 1959–61 than in the 1950s whereas no change was apparent at other ages (Fig. 6). However, in 1950 only one laryngeal cancer death occurred below the age of 55; hence no conclusions can be drawn on mortality changes at younger ages.

In females the age-adjusted laryngeal cancer mortality rates were similar in Polish-born and native white Americans in 1959–61, being slightly higher than the 1950 rates for Polish-born Americans (Table II); these rates are based on small numbers.

### DISCUSSION

Cancers of the upper alimentary tract and the larynx share at least two aetiological factors, namely tobacco and alcohol. Both these agents increase distinctly the risk of cancers of the buccal cavity and pharynx (except for salivary gland tumours and nasopharyngeal cancer), oesophagus and larynx (*Smoking and Health*, 1964). This association has also been demonstrated in Poland, at least for tobacco smoking (Staszewski, 1960, 1969).

The relative importance of tobacco and alcohol varies for the discussed cancer sites. Thus the association with alcohol appears to be stronger for oesophageal, hypopharyngeal, tongue and extrinsic laryngeal cancers, whereas that with smoking is stronger for intrinsic laryngeal cancer (Flamant *et al.*, 1964; *The Health Consequences of Smoking*, 1971).

Exposure to sunlight increases the risk of cancer of the lip but is unlikely to be involved in the development of the other discussed cancers.

How can our findings be related to the suspected environmental factors?

The unremarkable position of Poland in relation to other countries suggests that no unusual (quantities of) carcinogens are operating in Poland. The relatively high and increasing incidence of laryngeal cancer may be due to the high and increasing cigarette consumption (1063 cigarettes per capita in 1950, 1539 in 1960 and 2062 in 1970); other forms of tobacco consumption are infrequent in Poland.

Alcohol consumption in Poland is not very high (equivalent to 4·6 litres of pure alcohol per capita in 1967, compared with 17·6 in France, 6·3 in the United States and 5·5 in Great Britain), which may account for the lower position of Poland for cancers of the buccal cavity, pharynx and oesophagus. Also, the structure of that consumption may be of importance: Polish consumption of wine (5 litres per capita in 1967) and of beer (28 litres) is low, and 65% of alcohol* is consumed as liquor (vodka) (Gus, 1970).

The particular shape of the mortality-by-age curve of laryngeal cancer resembles the lung cancer curve shape, which has been demonstrated to be due to a summation of mortality of successive cohorts, each of whom experiences higher mortality than the older one (*Smoking and Health*, 1964). This indicates increasing exposure of the successive cohorts

* Or the equivalent to 3·0 litres of pure alcohol; Poland has the second highest liquor consumption among the 18 compared countries.

to a carcinogen (probably cigarette smoking), and suggests that the increase in laryngeal cancer incidence in Poland may extend into older age groups in the future.

Before looking for explanations for our findings related to Polish migrants, these observations will be compared with the other published migration studies. Haenszel (1961) compared cancer mortality in 1950 among migrants from 6 European countries to the United States with that in their countries of birth, and also with that among native white Americans. Male migrants from 5 of these countries (Italy, Germany, Sweden, Norway and Ireland, the exception being England and Wales) exhibited, for cancers of the buccal cavity and pharynx and of oesophagus,* distinctly higher mortality rates than in their countries of birth, and for oesophageal cancer also higher than among native white Americans (the migrants from England and Wales also displayed higher oesophageal cancer mortality rates than native Americans). Polish-born Americans conformed to these findings with the 5 countries. For females, on the other hand, mortality from both cancers was lower among the migrants than in their countries of origin, but this was not true for Polish migrants. In another set of comparisons of mortality of foreign-born from 12 countries, Polish males had the highest rates for oesophageal cancer and also for lung and stomach cancers.

In a study of patients admitted to Rosswell Park Memorial Institute, Buffalo, N.Y., from 1945 through 1956, Graham *et al.* (1963) reported that " Polish–American men had higher risks of lung, gastric, and particularly oesophageal cancer than did other foreign-born and native-born persons ". Data presented in that paper suggest also higher risks in Polish–American men, compared with other migrant groups, for cancer of the larynx, buccal cavity and pharynx (for the latter site, Italian-born men displayed a higher risk). Also, Polish–American women appeared to have a higher risk of buccal cavity cancer than other foreign-born except Italian. No data on oesophageal cancer in women are presented in that paper.

Time trends in cancer risks of a migrant group have been reported only for the Japanese migrants to the United States (Haenszel and Kurihara, 1968). Among males a decrease was observed for mortality from cancers of the buccal cavity, pharynx and oesophagus between 1949–52 and 1959–62, similar to that observed in Polish-born American males over a similar period. The number of cases of laryngeal cancer (9 males), and of female cases among Japanese migrants, was too small for reliable comparisons.

The observed large shifts in the migrant's cancer risk suggest the operation of environmental rather than of genetic factors. In this connection it is relevant to recall the changes in the risk of lung cancer which have been observed in male Polish migrants to the United States. In 1950 lung cancer mortality among these migrants was distinctly higher than either in Poland or in native white Americans (Staszewski and Haenszel 1965), and in 1959–61 this contrast had diminished but was still far from disappearing (Staszewski, in the press), as in the case of oesophageal and laryngeal cancers. For cancers of the buccal cavity and pharynx this contrast was smaller in 1950 and had disappeared by 1959–61.

An increased risk of lung cancer in native white Americans who migrated from farms to urban areas of the United States has been reported (Haenszel, Loveland and Sirken, 1962; Haenszel and Taeuber, 1964). It seems likely that a similar migration effect operated in Polish migrants, most of whom were born in farming rural areas of Poland and settled in urban areas of the United States. Such a migration effect may also have operated for the other discussed cancers.

* Laryngeal cancer has not been analysed in this study.

The intriguing decrease in the mortality from lung cancer, and also from cancers of the larynx, oesophagus, buccal cavity and pharynx, observed between 1950 and 1959–61 in the age groups below 65 may be due to differences between different groups of migrants. Thus, the " old " migration came predominantly from the poor, rural, farming areas of Poland. As relatively few migrated from these areas as children and adolescents, and migration from Poland to the United States was virtually stopped for some years around 1924, a substantial proportion of the younger age groups is made by the " new " influx of migrants who arrived in the United States during and after the Second World War, and thus was partly not included in the 1950 U.S. census and mortality data. In contrast to the " old ", this " new " migration, caused by the 1939–45 war, originated mainly from a different part of society: namely from higher socioeconomic classes and mostly from large cities.

The decrease in lung cancer mortality observed in the younger age groups of Polish-born Americans could thus be due to the increased proportion of " new " migrants with different characteristics, such as possibly a lower cigarette consumption, or to the lack of migration effect because they were predominantly from urban and not rural areas.

The shift in oesophageal cancer risk observed in Polish male migrants was even larger than that in lung cancer risk. This might be due to possible changes in alcohol consumption. It is possible that an increase in alcohol consumption in these migrants above the level prevailing in both their country of origin and of adoption may have been promoted by difficulties in adaptation to their new environment and by their losing the old social restraints before acquiring new ones. After a longer stay in the new environment, adaptation to it might have helped to reduce the alcohol consumption to a lower level. Such explanation is indirectly supported also by the lack of increase in the risk of oesophageal cancer (and also of cancer of the buccal cavity and pharynx) in male English migrants who, according to Bogue (1959), seemed to encounter fewer problems with respect to adjustment or assimilation in the United States.

Verification of these speculations would require much more detailed knowledge of the habits and customs of Polish migrants, of their smoking habits and alcohol consumption, residence history (especially age at migration), occupational history, etc.

Besides such investigations, in the form of case control and population studies, continued collection of data on cancer incidence is required. Evaluation of further time trends calls for more recent data on mortality among Polish-born Americans. The incidence data, soon to be available from the American 1959–71 cancer survey and from some cancer registries, will also be valuable.

Unpublished tabulations of cancer mortality in Poland were kindly provided by the Department of Vital Statistics and Demographic Studies of the Central Statistical Office in Warszawa.

The National Center for Health Statistics, Washington, D.C., kindly permitted the access to the unpublished data on cancer mortality among the Polish-born and native-white Americans.

A part of the technical work, such as computations and drawings, was financed by the PL-480 Agreement 05-009-01, sponsored by the National Cancer Institute, National Institutes of Health, Bethesda, Maryland.

### REFERENCES

Bogue, D. J. (1959) *The Population of the United States*. New York. N.Y.: The Free Press of Glencoe.

Doll, R., Muir, C. S. & Waterhouse, J. A. H. (1970) Eds. *Cancer Incidence in Five Continents*, Vol. II. Geneva: UICC.

Flamant, R., Lasserre, O., Lazar, P., Leguerinais, J., Denoix, P. & Schwartz, D. (1964) Differences in Sex Ratio according to Cancer Site and Possible Relationship with Use of Tobacco and Alcohol. Review of 65,000 Cases. *J. natn. Cancer Inst.*, **32**, 1309.

GRAHAM, S., LEVIN, M. L., LILIENFELD, A. M. & SHEEHE, P. (1963) Ethnic Derivation as Related to Cancer at Various Sites. *Cancer, N.Y.*, **16**, 13.

GUS (Główny Urząd Statystyczny) (1970) *Napoje alkoholowe.* Warszawa: GUS.

HAENSZEL, W. (1961) Cancer Mortality among the Foreign-born in the United States. *J. natn. Cancer Inst.*, **26**, 37.

HAENSZEL, W. & KURIHARA, M. (1968) Studies of Japanese Migrants. I. Mortality from Cancer and Other Diseases among Japanese in the United States. *J. natn. Cancer Inst.*, **40**, 43.

HAENSZEL, W., LOVELAND, D. B. & SIRKEN, M. G. (1962) Lung Cancer Mortality as Related to Residence and Smoking Histories. I. White Males. *J. natn. Cancer Inst.*, **28**, 947.

HAENSZEL, W. & TAEUBER, K. R. (1964) Lung Cancer Mortality as Related to Residence and Smoking Histories. II. White Females. *J. natn. Cancer Inst.*, **32**, 803.

KOSZAROWSKI, T., KOŁODZIEJSKA, H., GADOMSKA, H., STASZEWSKI, J., WIECZORKIEWICZ, A., WRONKOWSKI, Z., WARDA, B. & KAREWICZ, Z. (1972) Cancer Registry Report in Selected Areas of Poland, 1965–70. *Organization of Cancer Control in Poland.* Warsaw: Polish Medical Publishers.

SEGI, M. & KURIHARA, M. (1972) *Cancer Mortality for Selected Sites in* 24 *Countries.* No. 6 (1966–67). Japan Cancer Society.

SMOKING AND HEALTH (1964) Report of the Advisory Committee to the Surgeon General of the Public Health Service. Washington, D.C.: Public Health Service Publication No. 1103.

STASZEWSKI, J. (1960) Smoking and Cancer in Poland. *Br. J. Cancer*, **14**, 419.

STASZEWSKI, J. (1964) Cancer in Poland in 1959. *Br. J. Cancer*, **18**, 1.

STASZEWSKI, J. (1969) Smoking and Cancer of the Alimentary Tract in Poland. *Br. J. Cancer*, **23**, 247.

STASZEWSKI, J. (1974) *Epidemiology of Cancer of Selected Sites in Poland and Polish Migrants. A Comparative Study.* Cambridge, Mass.: Ballinger Publishing Company. In the press.

STASZEWSKI, J. & HAENSZEL, W. (1965) Cancer Mortality among the Polish-born in the United States. *J. natn. Cancer Inst.*, **35**, 291.

THE HEALTH CONSEQUENCES OF SMOKING. (1971) A Report to the Surgeon General, Washington, D.C.: U.S. Department of Health, Education and Welfare, Public Health Service.


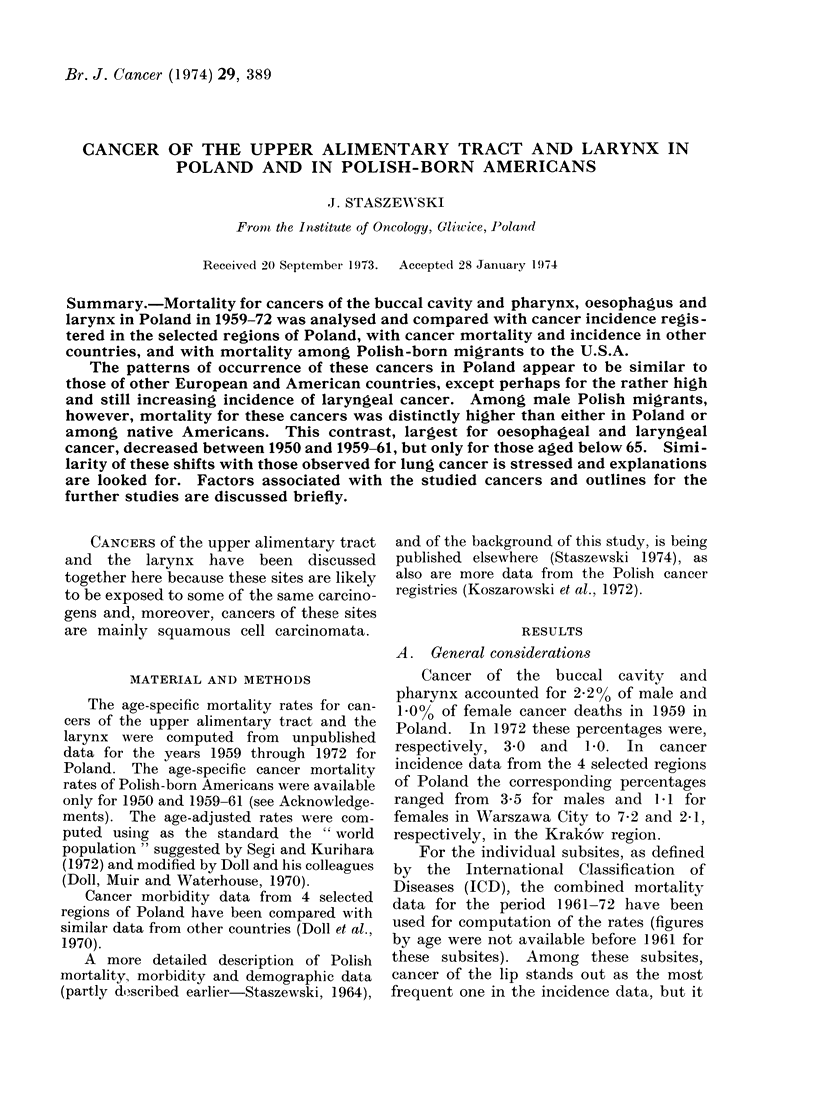

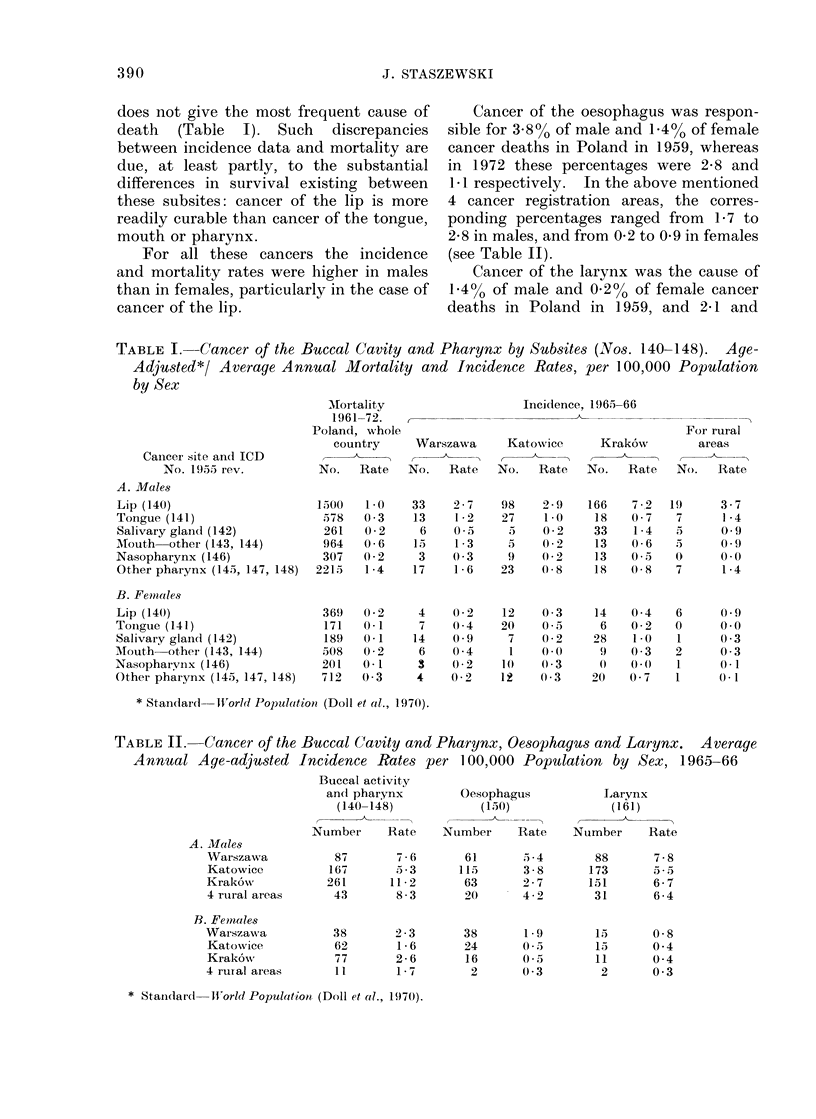

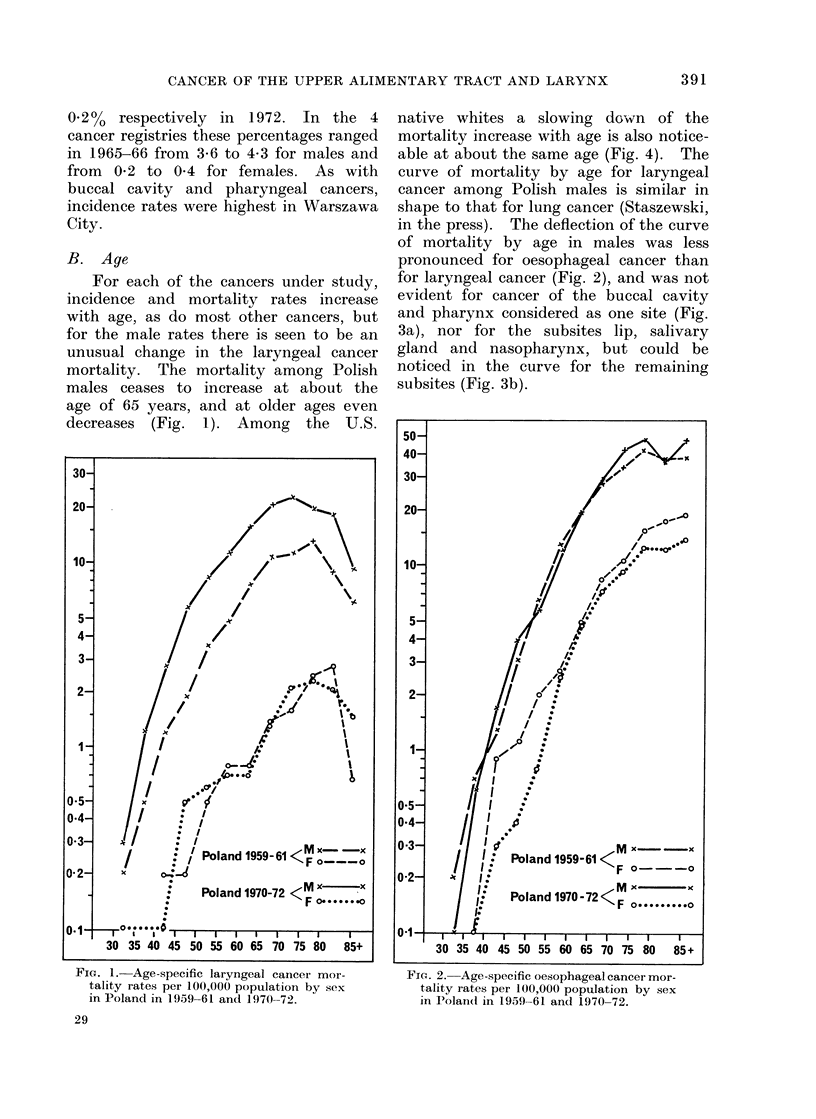

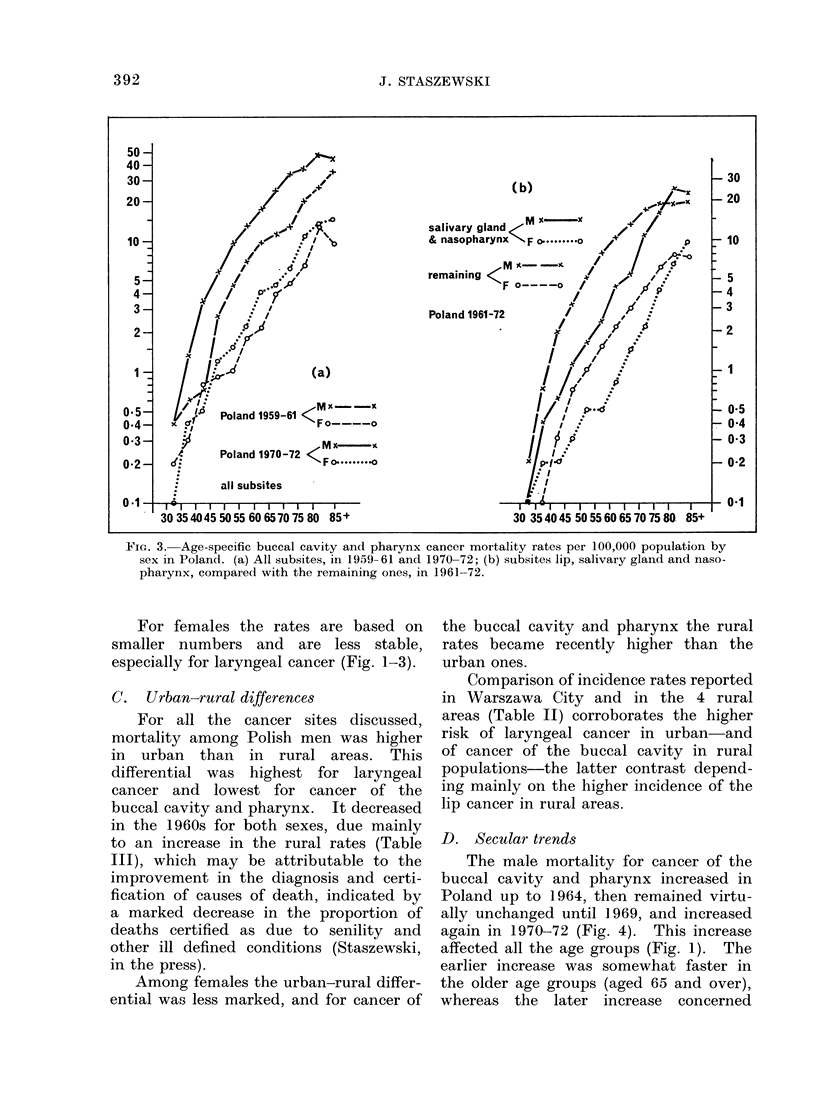

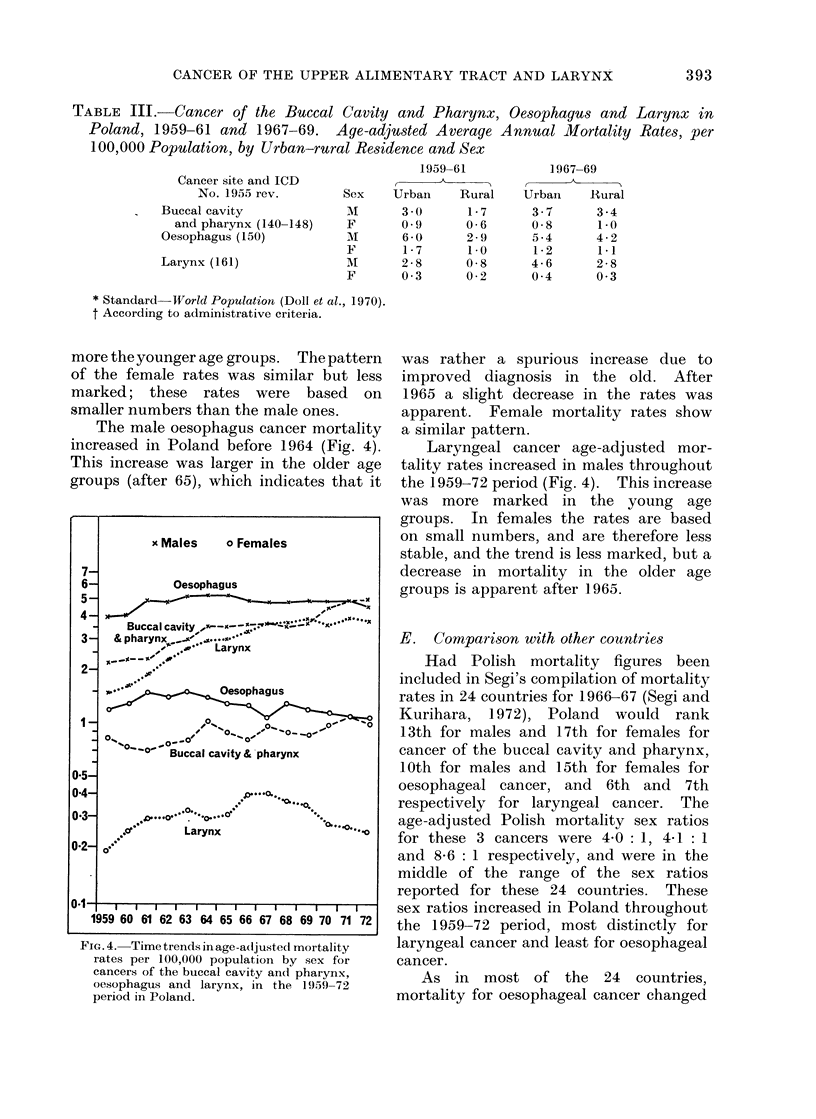

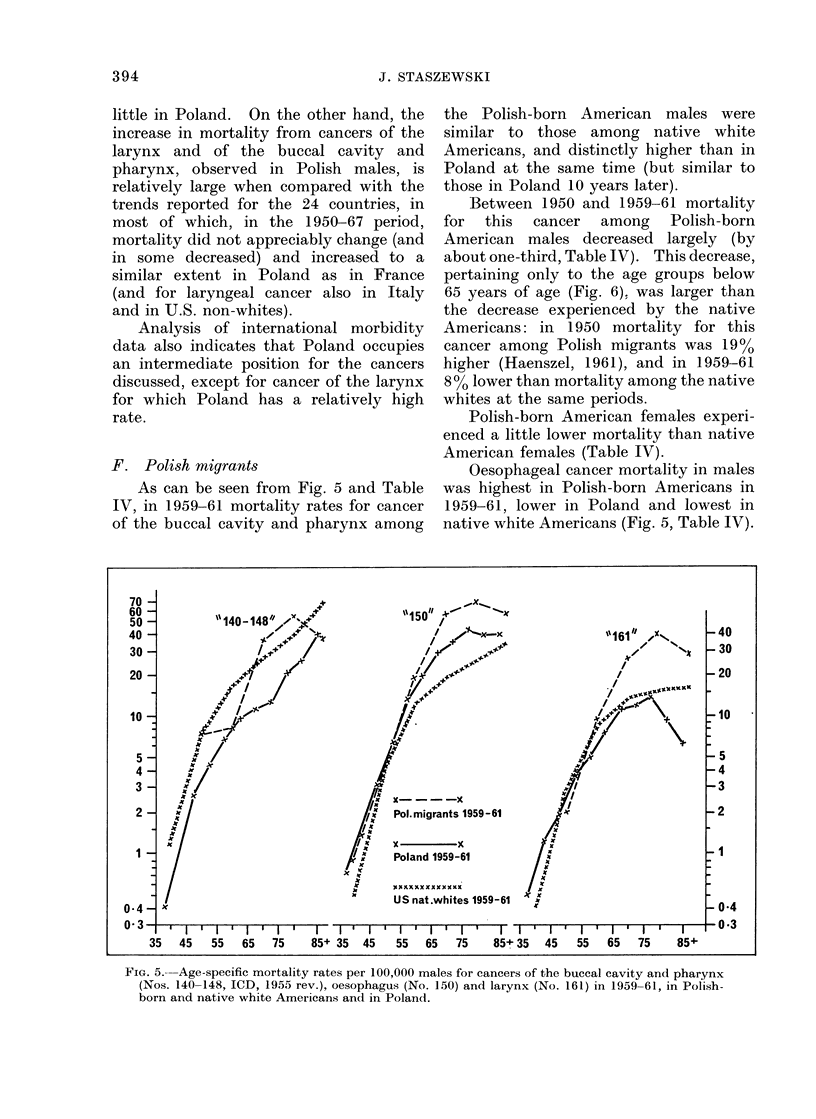

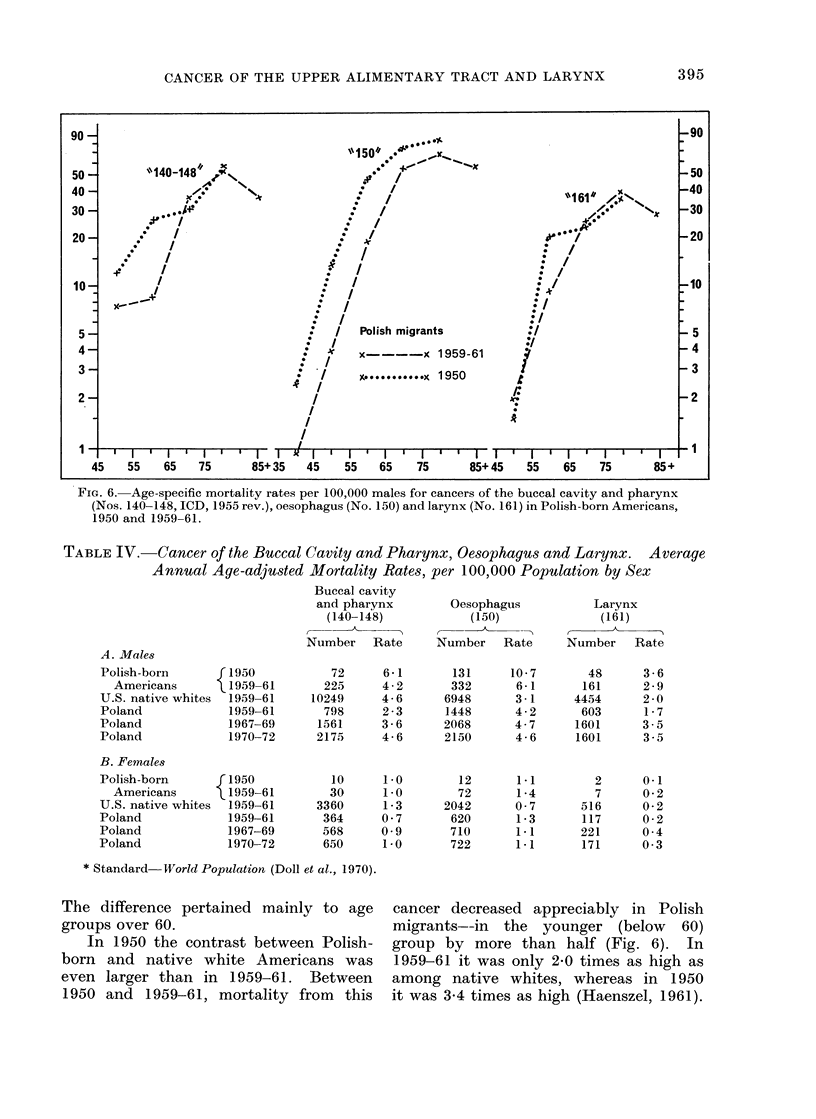

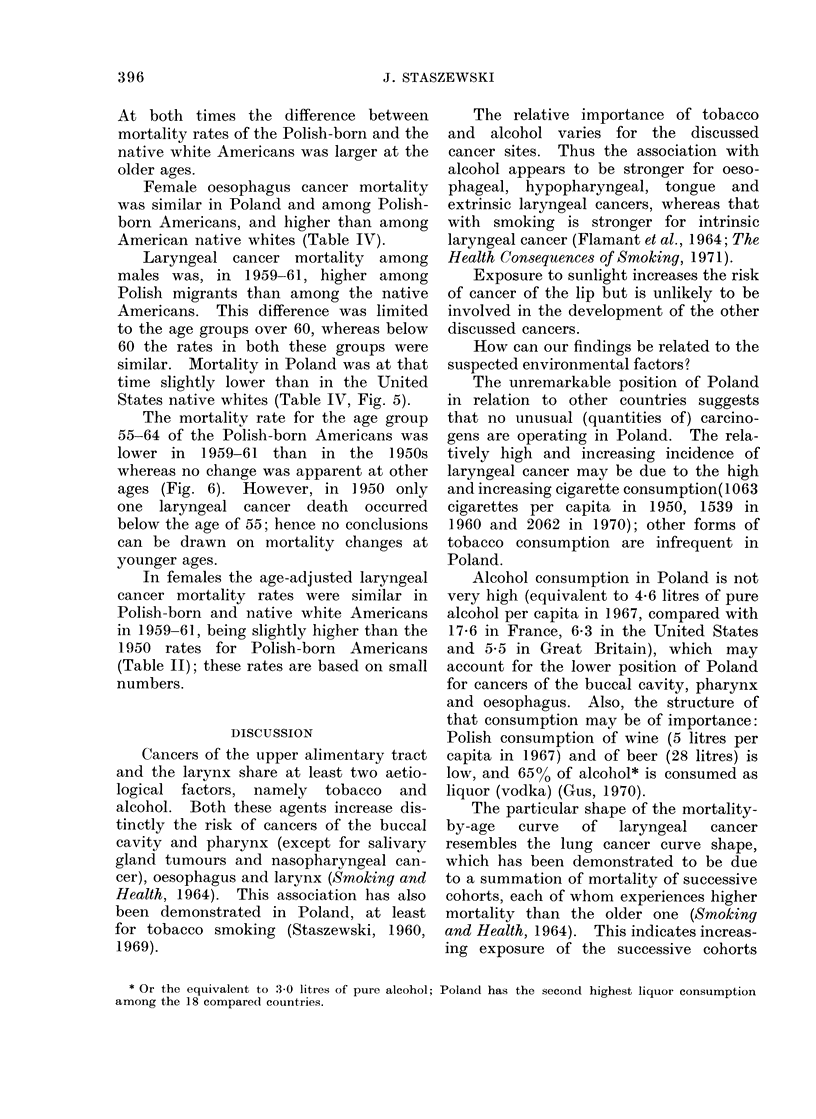

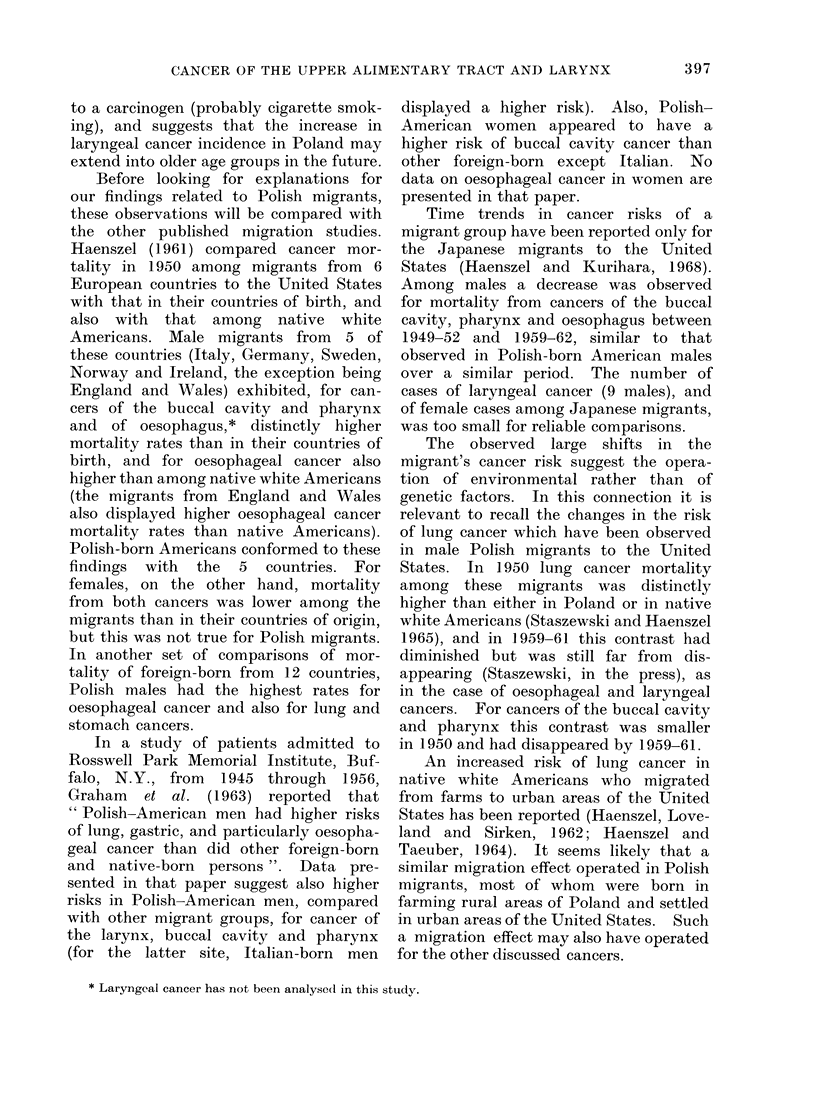

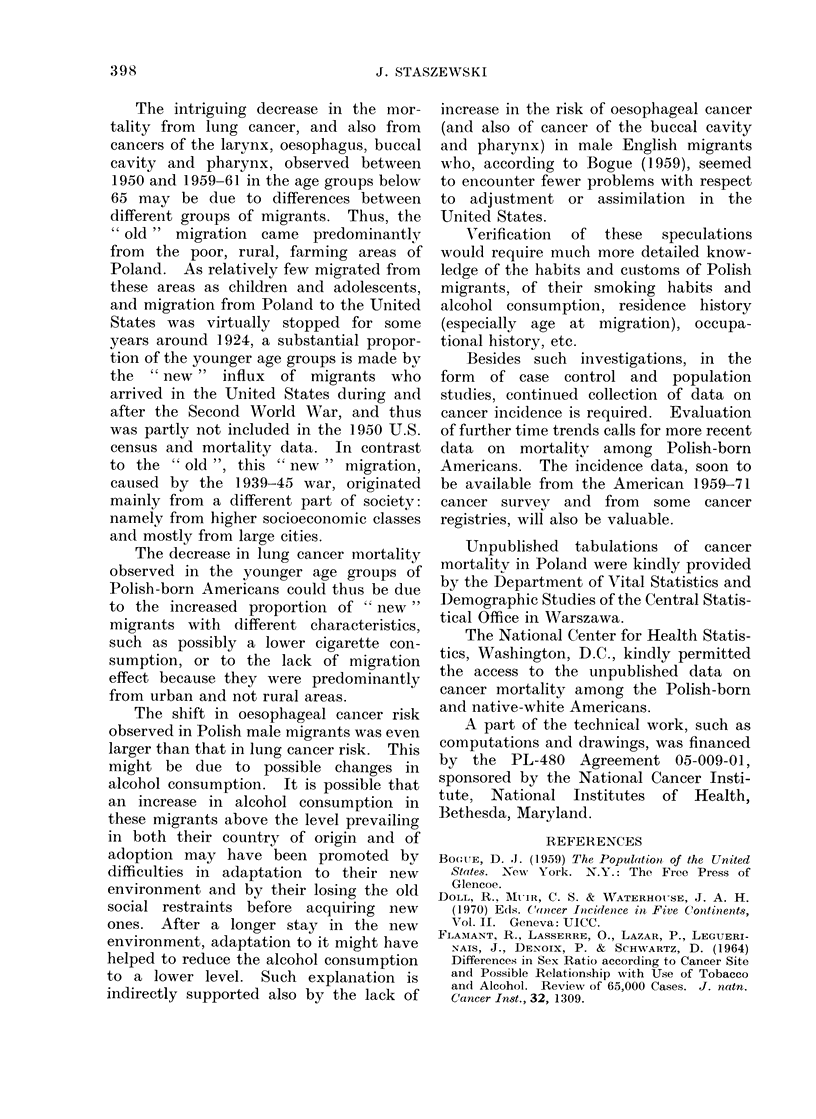

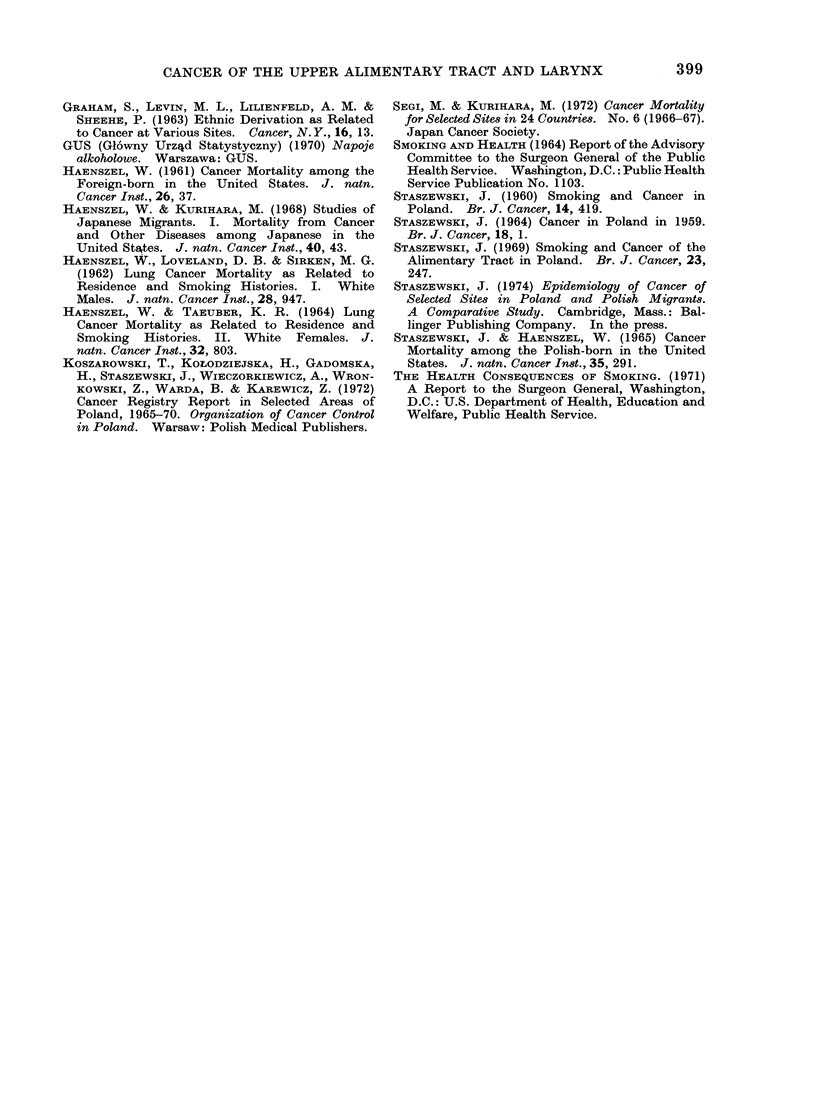


## References

[OCR_01534] FLAMANT R., LASSERRE O., LAZAR P., LEGUERINAIS J., DENOIX P., SCHWARTZ D. (1964). DIFFERENCES IN SEX RATIO ACCORDING TO CANCER SITE AND POSSIBLE RELATIONSHIP WITH USE OF TOBACCO AND ALCOHOL. REVIEW OF 65,000 CASES.. J Natl Cancer Inst.

[OCR_01542] GRAHAM S., LEVIN M. L., LILIENFELD A. M., SHEEHE P. (1963). Ethnic derivation as related to cancer at various sites.. Cancer.

[OCR_01561] HAENSZEL W., LOVELAND D. B., SIRKEN M. G. (1962). Lung-cancer mortality as related to residence and smoking histories. I. White males.. J Natl Cancer Inst.

[OCR_01596] STASZEWSKI J. (1964). CANCER IN POLAND IN 1959.. Br J Cancer.

[OCR_01611] Staszewski J., Haenszel W. (1965). Cancer mortality among the Polish-born in the United States.. J Natl Cancer Inst.

[OCR_01600] Staszewski J. (1969). Smoking and cancer of the alimentary tract in Poland.. Br J Cancer.

